# Identification of rhabdoviral sequences in oropharyngeal swabs from German and Danish bats

**DOI:** 10.1186/s12985-014-0196-x

**Published:** 2014-11-25

**Authors:** Melina Fischer, Conrad M Freuling, Thomas Müller, Juliane Schatz, Thomas Bruun Rasmussen, Mariann Chriel, Anne Balkema-Buschmann, Martin Beer, Bernd Hoffmann

**Affiliations:** Institute of Diagnostic Virology, Friedrich-Loeffler-Institut, Südufer 10, D-17493 Greifswald-Insel Riems, Germany; Institute of Molecular Virology and Cell Biology, Friedrich-Loeffler-Institut, Südufer 10, D-17493 Greifswald-Insel Riems, Germany; DTU Vet, Technical University of Denmark, Lindholm, DK-4771 Kalvehave, Denmark; DTU Vet, Technical University of Denmark, DK-1870 Frederiksberg C, Denmark; Institute of Novel and Emerging Infectious Diseases, Friedrich-Loeffler-Institut, Südufer 10, D-17493 Greifswald-Insel Riems, Germany

**Keywords:** Pan-lyssavirus PCR, *Rhabdoviridae*, Dimarhabdovirus supergroup

## Abstract

**Background:**

In the frame of active lyssavirus surveillance in bats, oropharyngeal swabs from German (N = 2297) and Danish (N = 134) insectivorous bats were investigated using a newly developed generic pan-lyssavirus real-time reverse transcriptase PCR (RT-qPCR).

**Findings:**

In total, 15 RT-qPCR positive swabs were detected. Remarkably, sequencing of positive samples did not confirm the presence of bat associated lyssaviruses but revealed nine distinct novel rhabdovirus-related sequences.

**Conclusions:**

Several novel rhabdovirus-related sequences were detected both in German and Danish insectivorous bats. The results also prove that the novel generic pan-lyssavirus RT-qPCR offers a very broad detection range that allows the collection of further valuable data concerning the broad and complex diversity within the family *Rhabdoviridae*.

## Findings

In the frame of active lyssavirus surveillance in bats [1], oropharyngeal swabs from 2297 German bats (sampled during May 2010 and September 2013) and 134 Danish bats (sampled during August to September 2013) were investigated, using a novel generic pan-lyssavirus real-time reverse transcriptase PCR (RT-qPCR) [2]. While all of those were previously tested negative by specific real-time RT-PCRs for the detection of *European bat lyssavirus* type 1 and 2 as well as *Bokeloh bat lyssavirus,* in total, 15 swabs from bats originating from the Northeast of Germany (12) and Central Jutland in Denmark (3) had Cq values between 33.5 and 44.6 when using the assay targeting a conserved region on the polymerase (L) gene [2].

Sequencing of those amplicons revealed nine distinct rhabdovirus-related sequences (GenBank accession numbers: KJ614421 - KJ614427, KJ873057, KJ873058) outside of the lyssavirus genus. The remaining six amplicons were identical to one of the nine sequences. Nucleotide BLAST [3] analysis showed nucleotide identities ranging from 75-86% compared to previously described members of the *Rhabdoviridae* family within the dimarhabdovirus supergroup (dipteran-mammal associated rhabdovirus [4]).

The partial L gene sequences from virus samples 26907 [KJ614425, *Myotis nattereri*], 27676 [KJ614426, *Eptesicus serotinus*] and RV-DK2 [KJ873058, *Myotis daubentonii*] however, showed the highest homology with less than 80% with *Isfahan virus* [AJ810084], *Kotonkan virus* [HM474855] and *Long island tick rhabdovirus* [KJ396935]. These rhabdoviruses were isolated from the sandfly *Phlebotomus papatasi*, from *Culicoides* sp. and from *Amblyomma americanum*, respectively. The sequences of the German samples 26530 [KJ614421, *Myotis nattereri*], 26855 [KJ614422, *Myotis mystacinus*], 26873 [KJ614423, *Eptesicus nilssonii*], 26876 [KJ614424, *Eptesicus nilsonii*] and 29130 [KJ614427, *Myotis bechsteinii*] as well as the Danish sample RV-DK1 [KJ873057, *Myotis daubentonii*] showed the highest identities with less than 75% for different strains of *Wongabel virus*, *Mossuril virus* or *Bovine ephemeral fever virus*.

**Figure 1:**
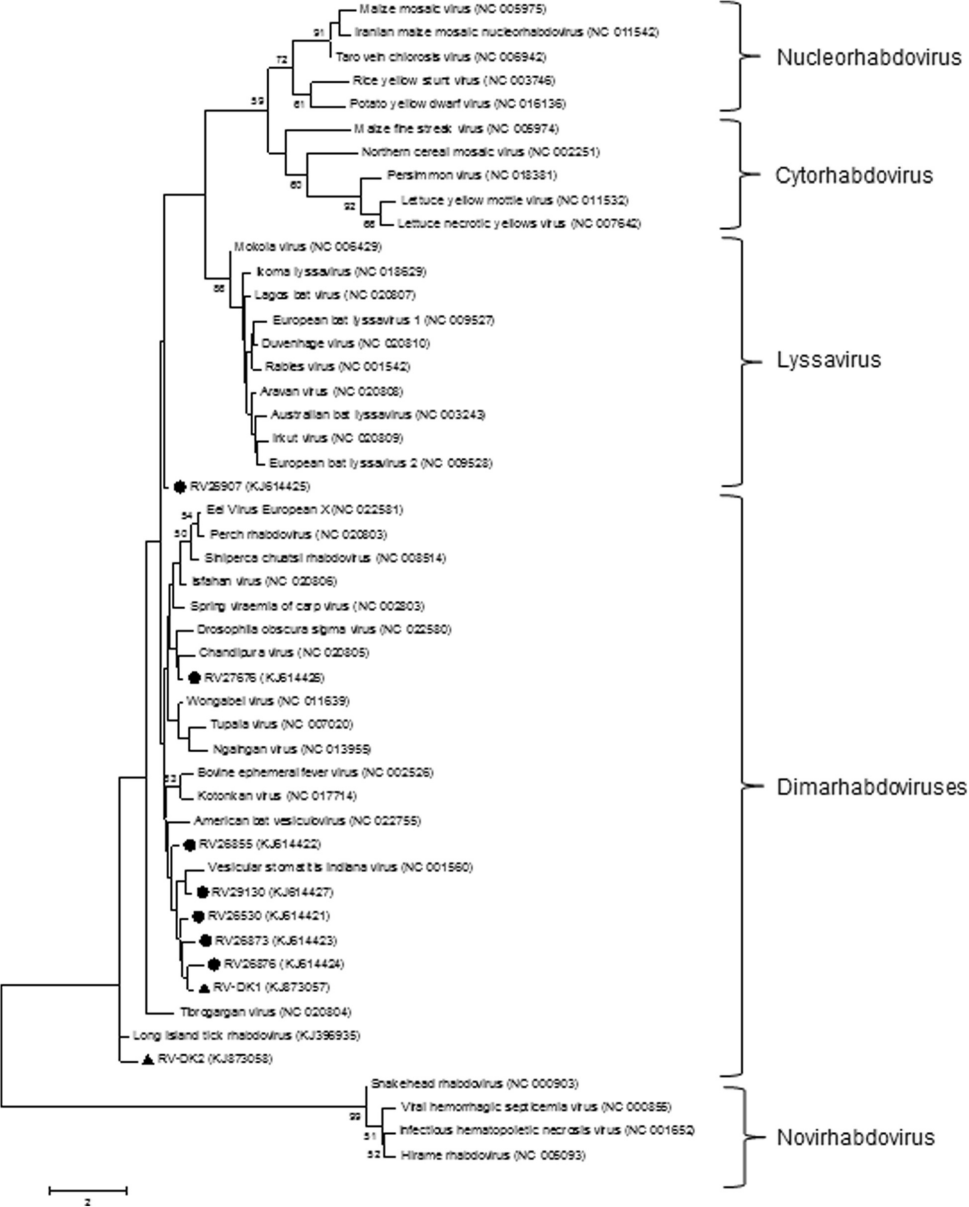
**Phylogenetic analysis of novel rhabdovirus-related sequences obtained from active surveillance of German (**  **) and Danish bats (▲).** The evolutionary history was inferred by using the Maximum Likelihood method based on the Kimura 2-parameter model [5]. For the phylogenetic analysis the RefSeq sequence information for *Rhabdoviridae* available in GenBank and the Long Island tick rhabdovirus were used. Bootstrap values higher than 50% were depicted. The tree with the highest log likelihood (−4926,7333) is shown. The percentage of trees in which the associated taxa clustered together is shown next to the branches. Initial tree(s) for the heuristic search were obtained automatically by applying Neighbor-Joining and BioNJ algorithms to a matrix of pairwise distances estimated using the Maximum Composite Likelihood (MCL) approach, and then selecting the topology with superior log likelihood value. A discrete Gamma distribution was used to model evolutionary rate differences among sites (5 categories (+G, parameter = 0,4501)). The tree is drawn to scale, with branch lengths measured in the number of substitutions per site. The analysis involved 49 nucleotide sequences. All positions containing gaps and missing data were eliminated. There were a total of 143 positions in the final dataset. Evolutionary analyses were conducted in MEGA6 [6].

A preliminary phylogenetic analysis using RefSeq sequence information from the family *Rhabdoviridae* in GenBank confirmed the affinity of all nine distinct rhabdovirus-related sequences among the dimarhabdovirus supergroup (Figure 1). The differences between the BLAST search and the phylogenetic relationship is based on the fact, that only a limited number of sequence data were integrated in the phylogenetic analysis. Furthermore the BLAST search and the phylogenetic analysis use clearly different algorithms for the identification of sequence similarities and phylogenetic relations, respectively. Nevertheless, the pan-lyssavirus RT-qPCR targets a highly conserved genome region in the polymerase (L) gene and was used as an independent alternative to the frequently used nucleoprotein (N) gene targeting assays. The fragment size of 172 bp is very short and suboptimal for conclusive sequence analyses. Thus the generation of further sequence information, including whole genome sequencing would be valuable for further phylogenetic analyses of these samples. However, the available sample material and the viral genome load were very limited.

For the German samples, based on the generated sequence data, specific real-time RT-PCRs were developed and applied (Table 1), using the OneStep RT-PCR Kit (Qiagen, Hilden, Germany). Briefly, 5.5 μl RNase-free water, 2.5 μl 5x OneStep RT-PCR Buffer, 0.5 μl OneStep RT-PCR Enzyme Mix, 0.5 μl dNTP Mix (10 mM each), 0.5 μl ResoLight Dye (Roche, Mannheim, Germany), 0.25 μl of each primer (20 pmol/μl) and 2.5 μl RNA template or RNase free water for the no template control (NTC) were used for each reaction. The following thermal program was applied: 1 cycle of 50°C for 30 min and 95°C for 15 min, followed by 45 cycles of 94°C for 30 s, 56°C for 30 s, and 72°C for 30 s. Subsequently melt curve analysis was performed (1 min 95°C, 1 min 55°C, increase 0.5°C per cycle for 10 s; 55-95°C). Obtained viral genome loads ranged between quantification cycle (C_q_) values of 26–36 (Table 1) indicating medium to low viral loads.

**Table 1:** **Oligonucleotides used for specific PCR systems and resulting C**_**q**_ **-values for rhabdoviral sequences of German bats**

**Assay**	**Primer**	Position (nt)*	**Sequence (5′-3′) in the L-gene**	**C**_**q**_ **- value**	**Reference**
Rhabdo 1	rhabdo-L1-F	6-139	GAGAGTATTTTGTTGTAACTGAGC	28.5	KJ614426
	rhabdo-L1-R		GTCTAGACCTTGTCCATTGGA		
Rhabdo2	rhabdo-L2-F	3-128	TGAGGGAGTATTTCGTGATGAC	30.3	KJ614425
	rhabdo-L2-R		GTCCGCTTGATGTTTCCAGC		
Rhabdo3	rhabdo-L3-F	4-147	GAGAGACTACTTTGTCATCACTG	28.6	KJ614422
	rhabdo-L3-R		TCGTAATCATCTAGACCTTGTCC		
Rhabdo4	rhabdo-L4-F	4-123	GAGGGATTACTTTGTAATAACTGAG	31.8	KJ614427
	rhabdo-L4-R		AGCCTTGGCCATTTGATGTATTC		
Rhabdo5	rhabdo-L5-F	3-147	TTCGCGACTACTTCGTCATAAC	25.9	KJ614424
	rhabdo-L5-R		TCGTAATTGTTTAATCCCTGTCC		
Rhabdo6	rhabdo-L6-F	1-134	GCTGAGGGATTACTTTGTAATTAC	34.6	KJ614421
	rhabdo-L6-R		ATCCTTGTCCATTTGACGTGTC		
Rhabdo7	rhabdo-L7-F	1-140	GTTACGAGATTACTTTGTGATCAC	35.8	KJ614423
	rhabdo-L7-R		TGTCCAGGCCTTGTCCATTG		

*Position according to reference.

Recently, highly sensitive sophisticated molecular techniques have been used to elucidate the role bats play in the epidemiology of rhabdoviruses. Our results corroborate previous studies that have revealed the presence of novel rhabdoviruses or rhabdoviral sequences in bats from Africa, America and Europe [7-13]. Similar to a previous study which detected rhabdoviral RNA in oropharyngeal swabs from Spanish bats using a generic nested RT-PCR [13], we were not able to decipher whether the viral sequences originated directly from the infected bats or from their arthropod/insect prey animals, because all positive samples were collected from different insectivorous bats. However, the fact that the majority of viruses with the highest sequence homology are rhabdoviruses of insects, suggests an arthropod origin for these novel sequences.

In conclusion, we were able to detect novel rhabdovirus-related sequences in oropharyngeal swabs obtained from German and Danish bats, using a generic pan-lyssavirus L-gene RT-qPCR. As indicated previously [2], this system offers an extremely broad detection range. Moreover, the PCR-assay allowed the collection of valuable data to increase our knowledge of the broad and complex diversity within the family *Rhabdoviridae*.

## Abbreviations

C_q_-value: Quantification cycle value
dimarhabdovirus: Dipteran-mammal associated rhabdovirus
L-gene: Polymerase gene
RT-qPCR: Real-time reverse transcriptase PCR
